# Supplementary material for: Characterization of Porcine Urinary Bladder Matrix Hydrogels from Sodium Dodecyl Sulfate Decellularization Method
Source: Polymers (Basel). 2020 Dec 16;12(12):3007. doi: 10.3390/polym12123007 (PMC7766358; doi:10.3390/polym12123007)
Supplement: Supplementary file 1 [file polymers-12-03007-s001.pdf]

polymers

MDPI

Supplementary Materials

Characterization of Porcine Urinary Bladder Matrix Hydrogels from Sodium Dodecyl Sulfate Decellularization Method

Chen-Yu Kao [1,2,*], Huynh-Quang-Dieu Nguyen [1,3] and Yu-Chuan Weng [4]

[1] Graduate Institute of Biomedical Engineering, National Taiwan University of Science and Technology, Taipei 10607, Taiwan
[2] Biomedical Engineering Research Center, National Defense Medical Center, Taipei 11490, Taiwan
[3] Graduate Institute of Applied Science and Technology, National Taiwan University of Science and Technology, Taipei 10607, Taiwan; d10822805@mail.ntust.edu.tw
[4] School of Medicine, National Defense Medical Center, Taipei 11490, Taiwan; 407010125@mail.ndmctsgh.edu.tw
* Correspondence: ckao@mail.ntust.edu.tw; Tel.: +886-2-2730-3676; Fax: +886-2-2730-3733

Received: 3 December 2020; Accepted: 15 December 2020; Published: date

The following are available online at www.mdpi.com/xxx/s1. Figure S1: SDS residual inside the decellularized scaffold. Table S1: Turbidimetric analysis of the UBM hydrogel gelation kinetics, using PAA decellularization method. Table S2: Turbidimetric analysis of the UBM hydrogel gelation kinetics, using SDS decellularization method. Table S3: Constants from the viscosity measurements of pre-gel solutions from PAA group. Table S4: Constants from the viscosity measurements of pre-gel solutions from SDS group.

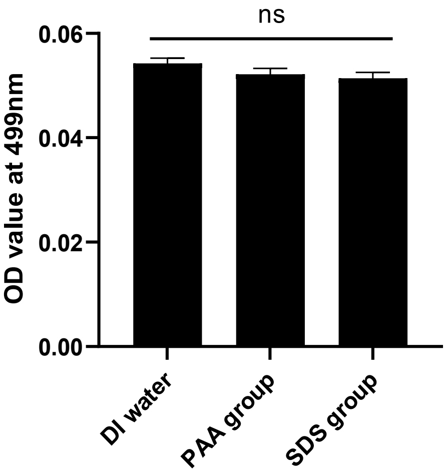

Figure S1. SDS residual inside the decellularized scaffold (n = 3). * Denotes a statistically significant difference.

Table S1. Turbidimetric analysis of the UBM hydrogel gelation kinetics, using PAA decellularization method (n = 4).

Hydrogel Concentration	Speed (min^{-1})	T_{lag} (min)	$T_{1/2}$ (min)
2 mg/mL	0.062 (±0.005)	10.72 (±0.57)	18.69 (±1.38)
4 mg/mL	0.063 (±0003)	10.97 (±0.58)	18.80 (±1.01)
6 mg/mL	0.057 (±0.002)	12.49 (±0.60)	21.78 (±0.40)
8 mg/mL	0.045 (±0.007)	12.64 (±0.19)	23.86 (±0.64)

Table S2. Turbidimetric analysis of the UBM hydrogel gelation kinetics, using SDS decellularization method (n = 4).

Hydrogel Concentration	Speed (min^{-1})	T_{lag} (min)	$T_{1/2}$ (min)
2 mg/mL	0.042 (±0.008)	13.88 (±0.80)	27.19 (±2.25)
4 mg/mL	0.042 (±0009)	15.26 (±1.11)	27.43 (±2.60)
6 mg/mL	0.032 (±0.002)	17.12 (±0.58)	32.23 (±0.35)
8 mg/mL	0.026 (±0.002)	19.62 (±0.80)	38.14 (±1.14)

Table S3. Constants from the viscosity measurements of pre-gel solutions from PAA group (n = 3).

Concentration (mg/mL)	k	n	r2
4	0.1521 ± 0.1154	−0.3590 ± 0.0078	0.9925 ± 0
6	1.4717 ± 0.0335	−0.5477 ± 0.005	0.9991 ± 0.0002
8	4.9812 ± 0.0584	−0.6740 ± 0.002	0.9997 ± 0

Table 4. Constants from the viscosity measurements of pre-gel solutions from SDS group (n = 3).

Concentration (mg/mL)	k	n	r2
4	0.6704 ± 0.0258	−0.5267 ± 0.0075	0.9994 ± 0.0002
6	2.2318 ± 0.0921	−0.623 ± 0.0052	0.9999 ± 0.0001
8	5.3492 ± 0.4491	−0.7 ± 0.0085	0.9998 ± 0.0002

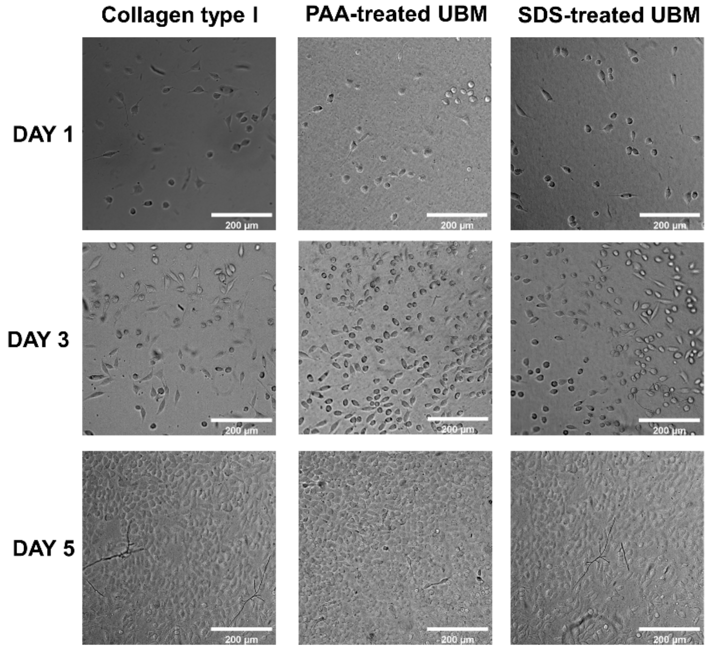

Figure S2. Optical images of cells cultured on hydrogel surface after 1, 3 and 5 days (scale bar: 200 µm).

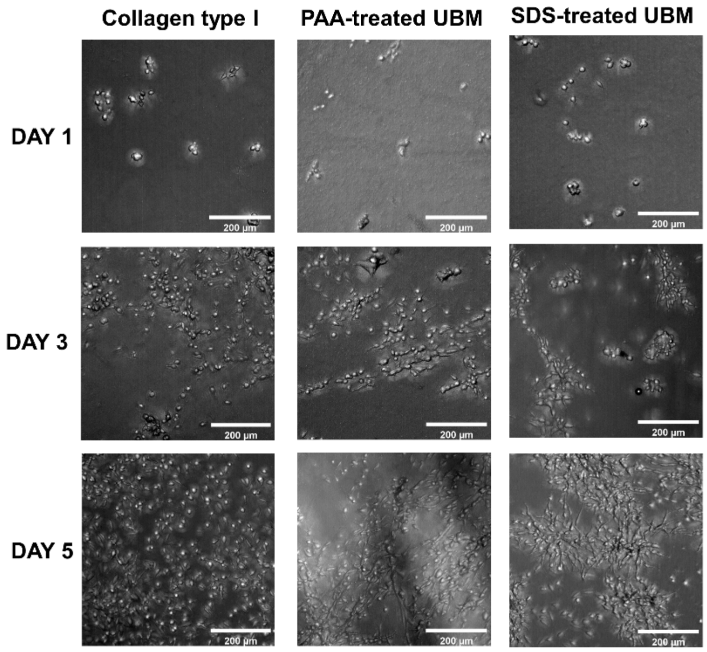

Figure S3. Optical images of cells cultured within hydrogel after 1, 3 and 5 days (scale bar: 200 μm).

Publisher's Note: MDPI stays neutral with regard to jurisdictional claims in published maps and institutional affiliations.

© 2020 by the authors. Submitted for possible open access publication under the terms and conditions of the Creative Commons Attribution (CC BY) license (http://creativecommons.org/licenses/by/4.0/).
